# Levosimendan Inhibits Peroxidation in Hepatocytes by Modulating Apoptosis/Autophagy Interplay

**DOI:** 10.1371/journal.pone.0124742

**Published:** 2015-04-16

**Authors:** Elena Grossini, Kevin Bellofatto, Serena Farruggio, Lorenzo Sigaudo, Patrizia Marotta, Giulia Raina, Veronica De Giuli, David Mary, Piero Pollesello, Rosalba Minisini, Mario Pirisi, Giovanni Vacca

**Affiliations:** 1 Laboratory of Physiology and Experimental Surgery, Department of Translational Medicine, University Eastern Piedmont “Amedeo Avogadro”, Via Solaroli 17, Azienda Ospedaliera Universitaria Maggiore della Carità, corso Mazzini 36, Novara, Italy; 2 Internal Medicine, Department of Translational Medicine, University Eastern Piedmont “Amedeo Avogadro”, Via Solaroli 17, Azienda Ospedaliera Universitaria Maggiore della Carità, corso Mazzini 36, Novara, Italy; Haassah Medical Center, ISRAEL

## Abstract

**Background:**

Levosimendan protects rat liver against peroxidative injuries through mechanisms related to nitric oxide (NO) production and mitochondrial ATP-dependent K (mitoK_ATP_) channels opening. However, whether levosimendan could modulate the cross-talk between apoptosis and autophagy in the liver is still a matter of debate. Thus, the aim of this study was to examine the role of levosimendan as a modulator of the apoptosis/autophagy interplay in liver cells subjected to peroxidation and the related involvement of NO and mitoK_ATP_.

**Methods and Findings:**

In primary rat hepatocytes that have been subjected to oxidative stress, Western blot was performed to examine endothelial and inducible NO synthase isoforms (eNOS, iNOS) activation, apoptosis/autophagy and survival signalling detection in response to levosimendan. In addition, NO release, cell viability, mitochondrial membrane potential and mitochondrial permeability transition pore opening (MPTP) were examined through specific dyes. Some of those evaluations were also performed in human hepatic stellate cells (HSC). Pre-treatment of hepatocytes with levosimendan dose-dependently counteracted the injuries caused by oxidative stress and reduced NO release by modulating eNOS/iNOS activation. In hepatocytes, while the autophagic inhibition reduced the effects of levosimendan, after the pan-caspases inhibition, cell survival and autophagy in response to levosimendan were increased. Finally, all protective effects were prevented by both mitoK_ATP_ channels inhibition and NOS blocking. In HSC, levosimendan was able to modulate the oxidative balance and inhibit autophagy without improving cell viability and apoptosis.

**Conclusions:**

Levosimendan protects hepatocytes against oxidative injuries by autophagic-dependent inhibition of apoptosis and the activation of survival signalling. Such effects would involve mitoK_ATP_ channels opening and the modulation of NO release by the different NOS isoforms. In HSC, levosimendan would also play a role in cell activation and possible evolution toward fibrosis. These findings highlight the potential of levosimendan as a therapeutic agent for the treatment or prevention of liver ischemia/reperfusion injuries.

## Introduction

Levosimendan has been suggested as a promising agent for protection against oxidative stress [1, 2]. Such beneficial effects would involve not only the improvement of hemodynamics, but also direct actions at tissue and cellular levels, which would be related to the mechanisms through which levosimendan could exert its effects. A large number of therapeutic agents against ischemia/reperfusion involves the regulation of mitochondrial function either through changes of membrane potential, reactive oxygen species (ROS) formation, or the modulation of K_ATP_ channels activity [3, 4]. In addition, experimental data suggest that nitric oxide (NO) could play a role in ischemia/reperfusion injury [5].

Regarding levosimendan, previously reported experimental *in vitro* and *in vivo* findings have suggested that it may protect heart, kidney and liver from apoptotic cell death by interfering with those mechanisms [5–8]. In particular, in anesthetized rats, intraportal infusion of levosimendan at the onset or reperfusion has recently been reported to reduce hepatocellular injury and allow a better-preserved liver integrity and function by interfering with oxidant/antioxidant status, Bax and caspase activation and the modulation of endothelial nitric oxide (eNOS)-dependent NO release, as well [8].

It is noteworthy that the beneficial effects of both *in vitro* and *in vivo* levosimendan were found to arise from its action on autophagy, a cellular degradation process which is involved in the turnover of dysfunctional organelles and proteins. Indeed, autophagy may either play a protective role, or contribute to cell damage acting as an alternative form of programmed cell death [9]. Interestingly, members of the Bcl-2 family proteins could modulate apoptosis/autophagy cross-talk so that autophagy may, up to a certain threshold, counteract apoptotic stimuli [10–12], as was also found in hepatocytes [13]. In this respect levosimendan was also able to protect cardiomyocytes against oxidative injuries through the modulation of the interplay between those cell death pathways [6].

Thus, the purpose of the present study was to examine in liver cells the effects of levosimendan on apoptosis and autophagy induced by oxidative stress and the involvement of mitoK_ATP_ channels and NO.

## Materials and Methods

Experiments were performed on rat hepatocytes obtained by collagenase perfusion of the liver taken from male Sprague-Dawley rats, following a method previously described [14] and approved by Comitato Etico per la Sperimentazione Animale (CESAPO) dell’Università del Piemonte Orientale “Amedeo Avogadro”. Cells were suspended in round-bottomed flasks rotating in a water bath maintained at 37°C in Krebs-Henseleit buffer (pH = 7.4; Sigma, Milan, Italy), supplemented with 12.5 mM HEPES (Sigma) under an atmosphere of 10% O_2_, 85% N_2_, and 5% CO_2_. For Griess study, cells were plated in 0.1% gelatin-coated 96-well plates in complete medium for 24 h and then maintained with Dulbecco Modified Eagle Medium (DMEM; Sigma), 0% foetal bovine serum (FBS; Sigma) supplemented with 1% penicillin-streptomycin-glutamine without red phenol (starvation medium; Sigma) overnight. For cell viability assay, mitochondrial membrane potential measurement and function, ROS detection and glutathione (GSH) content, cells were plated in 0.1% gelatin-coated dishes in complete medium and, at confluence, they were incubated with starvation medium overnight. The oxidative stress was generated using 200 μM H_2_O_2_ for 20 min in DMEM without FBS and red phenol. ROS detection, GSH levels and cell viability of hepatocytes were also examined by using as oxidative agent the superoxide anion donor, tert-butyl hydroperoxide (TBHP; 250 μM, Santa-Cruz Biotechnology, Inc, CA, USA) for 30 min. Control cells were treated with DMEM 0% FBS and phenol red only.

Some experiments were performed in immortalized human hepatic stellate cell line, LX-2, that was kindly provided by Prof. Scott Friedman (Mount Sinai Hospital, New York, NY, USA) and was cultured in DMEM containing 10% FBS, 1 mM glutamine, and 100 IU/ml streptomycin/penicillin [15]. Cultures were incubated at 37°C in a humidified atmosphere of 5% CO_2_ and the medium was changed every other day. For NO detection, cell viability, mitochondrial membrane potential and apoptosis/autophagy, LX-2 were maintained in starvation medium (0.2% FBS supplemented with 100 IU/ml penicillin-streptomycin-glutamine without red phenol) overnight. The oxidative stress was generated as described for hepatocytes.

### GSH quantification

The content of GSH was determined by using a commercial kit according to the manufacturer’s instructions (BioVision Inc., Milpitas, CA). Briefly, 1 x 10^6^ hepatocytes and LX-2, treated with 200 μM H_2_O_2_ or 250 μM TBHP (Santa-Cruz Biotechnology) in absence or presence of levosimendan (1 nM-100 nM-1000 nM) for 30 min, were homogenized on ice with 100 μl of ice cold Glutathione Assay Buffer. Thereafter, 60 μl of each homogenate was added to a pre-chilled tube containing perchloric acid (PCA) and vortexed for several seconds to achieve a uniform emulsion. After keeping on ice for 5 min, samples were spun for 2 min at 13000 G at 4°C and the supernatants were collected. Thereafter, 20 μl of ice cold 6N KOH was added to 40 μl of PCA preserved samples and after a further 2 min spinning at 13000 G at 4°C, 10 μl of the samples was transferred to 96-well plates where GSH was detected following manufacturer’s instructions and compared to standards. Samples and standards were read by spectrometer (BS1000 Spectra Count) at excitation/emission of 340 and 420 nm. GSH content was expressed as nmol/10^6^ cells.

### ROS quantification

The oxidation of 2,7-dichlorodihydrofluorescein diacetate (H2DCFDA) into 2,7-dichlorodihydrofluorescein (DCF) was used to assess ROS generation, following the manufacturer’s instructions (Abcam, Cambrigde, United Kingdom). Briefly, 1.5 x 10^5^ hepatocytes and LX-2 in 96-well plates were treated with 200 μM H_2_O_2_ or 250 μM TBHP (Santa-Cruz Biotechnology) in absence or presence of levosimendan (1 nM-100 nM-1000 nM) for 30 min. In some samples, hepatocytes were given the NOS blocker, Nω-nitro-L-arginine methyl ester (L-NAME, 10 mM; Sigma) or the mitochondrial K_ATP_ channels inhibitor, 5 hydroxydecanoate (5HD, 1 μM; Sigma) before H_2_O_2_ or TBHP (Santa-Cruz Biotechnology). After treatments, the reactions were stopped by removing medium and washing with PBS (Sigma) followed by staining with 10 μM H2DCFDA for 20 min at 37°C. The fluorescence intensity of H2DCFDA was measured at excitation/emission of 485 and 530 nm using a spectrometer (BS1000 Spectra Count).

### Cell viability

To determine cell viability, the In Vitro Toxicology Assay Kit MTT Based (Life Technologies Italia, Monza; Italy) was used. Hepatocytes and LX-2 (1 x 10^4^ cells) were cultured in 96-well plates in starvation medium. After treatments, the medium was removed and fresh culture medium without red phenol and FBS (Sigma) containing the 1% 3-[4,5-dimethylthiazol-2-yl]-2,5-diphenyl tetrazolium bromide (MTT) dye was added in 96-well plates containing the cells and incubated for 2 h at 37°C. Thereafter, the medium was removed and an MTT Solubilization Solution in equal volume to the original culture medium was added and mixed in a gyratory shaker until the complete dissolution of formazan crystals. Cell viability was determined by a spectrometer (BS1000 Spectra Count) and cell viability was calculated by comparing results with control cells (100% viable) [6].

The cells were treated with 200 μM H_2_O_2_ or 250 μM TBHP (Santa-Cruz Biotechnology) alone or in presence of 1 nM-100 nM-1000 nM levosimendan (SIMDAX) for 30 min. In some experiments, hepatocytes were treated with 5HD (1 μM; Sigma), L-NAME (10 mM; Sigma), the pan-caspases inhibitor, Benzyloxycarbonyl-Val-Ala-Asp (OMe) fluoromethylketone (Z-VAD.FMK, 25 mM; Sigma) [11], the autophagy inhibitor, 3-methyladenine (3-MA, 10 mM; Sigma) [11] and the autophagy activator, rapamycin (100 nM; Sigma) [16], before giving 1000 nM levosimendan. Those agents were also tested alone.

### NO production

The NO production was measured in cell culture supernatants using the Griess method (Promega, Milan, Italy). Hepatocytes and LX-2 (1 x 10^4^ cells) plated in 96-well plates in starvation medium were treated with 200 μM H_2_O_2_ alone or in presence of 1 nM-100 nM-1000 nM levosimendan (SIMDAX, Orion Corporation, Orionintie, Finland) administrated for 30 min. Those levosimendan concentrations were also tested in cells not subjected to peroxidation.

In addition, in some experiments, hepatocytes were treated with L-NAME (10 mM; Sigma), or 5HD (1 μM; Sigma), that were given before H_2_O_2_. The blockers and vehicles were also tested in the basal medium. At the end of stimulations, NO production in the sample’s supernatants was examined, as previously described [6, 17–19], by a spectrometer (570 nm; BS1000 Spectra Count, San Jose, CA, USA).

### Western blot

Western blot analysis was performed in hepatocytes and LX-2 at ~ 90% confluence in a 100 mm dishes in DMEM 0% FBS and red phenol (Sigma). After each stimulation, performed in hepatocytes with 200 μM H_2_O_2_ and the same agents used for cell viability and in LX-2 with 200 μM H_2_O_2_ alone or in presence of levosimendan (1 nM-100 nM-1000 nM), hapatocytes and LX-2 were washed with iced PBS 1X supplemented with 2 mM sodium orthovanadate (Sigma) and lysed in an iced-Ripa-buffer (10 mM Na2HPO4, 150 mM NaCl, 2 mM EDTA, 1% NP-40, 0.1% sodium dodecyl sulphate, 1% sodium deoxycholate, 50 mM sodium fluoride; Sigma) supplemented with 2 mM sodium orthovanadate (Sigma), 1:1000 phenylmethanesulfonylfluoride (PMSF; Sigma) and 1:100 protease inhibitors cocktail (Sigma). The extracted proteins were quantified by using bicinchoninic acid (BCA; Pierce, Rockford, IL, USA) and 40 μg from each lysate was resolved on 15% sodium dodecyl sulfate polyacrylamide electrophoresis gels (SDS-PAGE; Bio-Rad Laboratories, Hercules, CA, USA). After electrophoresis, proteins were transferred to polyvinylidene fluoride (PVDF) membranes (Bio-Rad Laboratories), which were incubated overnight at 4°C with specific antibodies: anti phospho-Bax (p-Bax; 1:500; Thr167; Assay Biotechnology Company, Sunnyvale, CA), anti Bax (1:500; Sigma), anti LC3I/II (1:1000; Santa-Cruz Biotechnology), anti phospho-Akt (p-Akt; 1:1000; Ser473, Cell Signalling Technologies), anti Akt (1:1000; Cell Signalling Technologies), in hepatocytes and LX-2. Anti-Beclin 1 (1:500, Santa-Cruz Biotechnology, Inc, CA, USA), anti Caspase 8 (1:1000, Cell Signalling Technologies, Beverly, MA, USA); anti phospo-Caspase 9 (p-Caspase 9; 1:300; against the active cleaved form of caspase-9; 1:500; Vinci-Biochem s.r.l., Vinci, Italy), anti Caspase 9 (Sigma), anti phospho-ERK1/2 (p-ERK1/2; 1:1000; Thr202/Tyr204, Cell Signalling Technologies), anti ERK1/2 (1:1000; Cell Signalling Technologies), anti phospo-eNOS (p-eNOS; 1:1000; Ser1177, Cell Signalling Technologies), anti eNOS (1:1000; Cell Signalling Technologies), anti iNOS (1:500; Santa-Cruz Biotechnology), in hepatocytes. The membranes were washed and then incubated with horseradish peroxidase-coupled goat anti rabbit IgG (Sigma) and horseradish peroxidase-coupled goat anti mouse IgG (Sigma) for 45 min, and were developed with a non-radioactive method using Western Lightning Chemiluminescence (PerkinElmer Life and Analytical Sciences, Waltham, MA). The protein expression was normalized through specific total protein and verified through β-actin (1:5000; Sigma) detection.

### Mitochondrial membrane potential measurement

For the measurement of mitochondrial membrane potential, after each stimulation performed with the same agents used for ROS quantification, the medium of 5 x 10^4^ hepatocytes and LX-2 plated in starvation medium was removed and the cells were incubated with JC-1 1x diluted in Assay Buffer 1x for 15 min at 37°C in a incubator following the manufacturer’s instruction (APO LOGIXTM JC-1; Invitrogen, Life Technologies Europe BV, Monza, Italy). The dye was dissolved in dimethylsulfoxide and the percentage of the organic solvent in the samples never exceeded 1% vol/vol. After the incubation, the cells were washed twice with Assay Buffer 1x and then the suspensions were transferred in triplicates to a black 96-well plates. The red (excitation 550 nm/emission 600 nm) and green (excitation 485 nm/emission 535 nm) fluorescence was measured using a fluorescence plate reader (BS1000 Spectra Count). To establish the cells undergoing apoptosis the ratio of red to green fluorescence was determined and expressed as %.

### Mitochondrial permeability transition pore (MPTP) opening

For the examination of MPTP opening, 5 x 10^4^ hepatocytes were plated in a 24-well plate in a complete medium for 4 h. After this time, the cells were maintained in starvation medium and then stimulated with the same agents used for ROS and mitochondrial membrane potential measurement. The sarcolemmal membrane of cells was permeabilized by using 10 μM of digitonin (Sigma) for 60 s and dissolved in an intracellular solution buffer (135 mM KCl, 10 mM NaCl, 20 mM HEPES, 5 mM pyruvate, 2 mM glutamate, 2 mM malate, 0.5 mM KH2PO4, 0.5 mM MgCl2, 15 mM 2,3-butanedione monoxime, 5 mM ethylene glycol tetra-acetic acid, 1.86 mMCaCl2; Sigma). The cells were loaded with 5 μM calcein/acetoxymethyl ester (AM; Sigma) for 40 min at 37°C to monitor the MPTP opening. After this time, the cells were washed with Tyrode solution (Sigma) for 10 min to remove the excess dye, and the calcein/AM fluorescence was measured by a fluorescence spectrometer with fluorescence excitation/emission of 488 and 510 nm, respectively. Pore-forming antibiotic alamethicin (10 μg/ml; Sigma) was applied to induce maximal calcein release from the mitochondrial matrix, and the minimum calcein fluorescence after alamethicin was regarded as 0% for the normalization of calcein fluorescence.

### Statistical analysis

All data were recorded using the Institution’s database. Statistical analysis was performed by using STATVIEW version 5.0.1 for Microsoft Windows (SAS Institute Inc, Cary NC, USA). Data were checked for normality before statistical analysis. One-way ANOVA followed by Bonferroni *post hoc* tests were used to examine changes among different groups of experiments. Results obtained in hepatocytes and LX-2 were expressed as means ± standard deviation (SD) of 5 or at least 3 independent experiments for each experimental protocol, respectively. A value of *P* <0.05 was considered as statistically significant.

## Results

### Levosimendan protects hepatocytes and LX-2 from peroxidation

**Fig 1:**
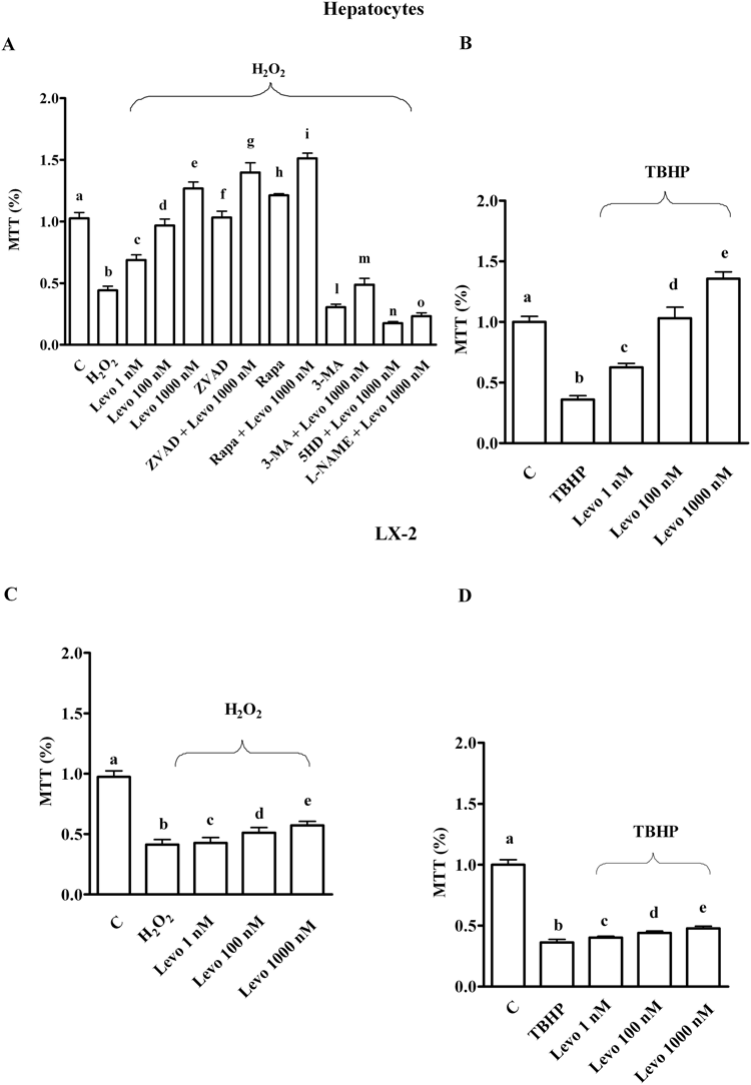
Effects of levosimendan on cell viability in hepatocytes and LX-2 that were subjected peroxidation. C = control; Levo = levosimendan; ZVAD = Z-VAD.FMK (Benzyloxycarbonyl-Val-Ala-Asp (OMe) fluoromethylketone), 25 mM; Rapa = rapamycin, 100 nM; 3-MA = 3-methyladenine, 10 mM; 5HD = 5 hydroxydecanoate, 1 μM; L-NAME = Nω-nitro-L-arginine methyl ester, 10 mM; MTT = 3-[4,5-dimethylthiazol-2-yl]-2,5-diphenyl tetrazolium bromide; TBHP = tert-butyl hydroperoxide, 250 μM. In A, b, c, d, e, f, g, h, i, l, m, n, o *P* < 0.05 vs a; c, d, e, f, g, h, i, l, m, n, o *P* <0.05 vs b; d, e *P* <0.05 vs c; e *P* <0.05 vs d; g, i, m, n, o *P* <0.05 vs e. In B, b, c, e *P* <0.05 vs a; c, d, e *P* <0.05 vs b; d, e *P* <0.05 vs c; e *P* <0.05 vs d. In C and D, b, c, d, e *P* <0.05 vs a; d, e *P* <0.05 vs b, c; e *P* <0.05 vs d. The results obtained in hepatocytes and LX-2 are expressed as means of 5 or 4 independent experiments (%) ± SD (indicated by the bars), respectively.

**Fig 2:**
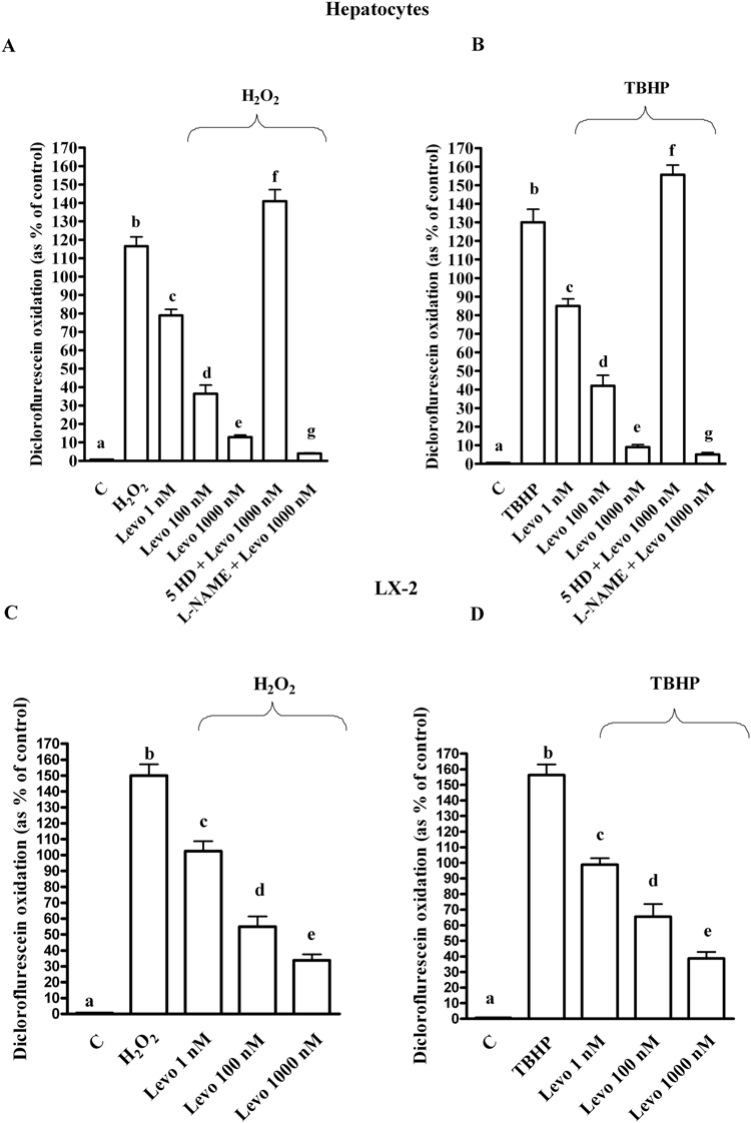
Effects of levosimendan against reactive oxygen species production measured as diclorofluorescein oxidation in hepatocytes and LX-2. In A and B, b, c, d, e, f, g *P* <0.05 vs a; c, d, e, f, g *P* <0.05 vs b; d, e *P* <0.05 vs c; d, f, g *P* <0.05 vs e. In C and D, b, c, d, e *P* <0.05 vs a; c, d, e *P* <0.05 vs b; d, e *P* <0.05 vs c; e *P* <0.05 vs d. Abbreviations are as in Fig 1. The results obtained in hepatocytes and LX-2 are expressed as means of 5 or 4 independent experiments (%) ± SD (indicated by the bars), respectively.

**Fig 3:**
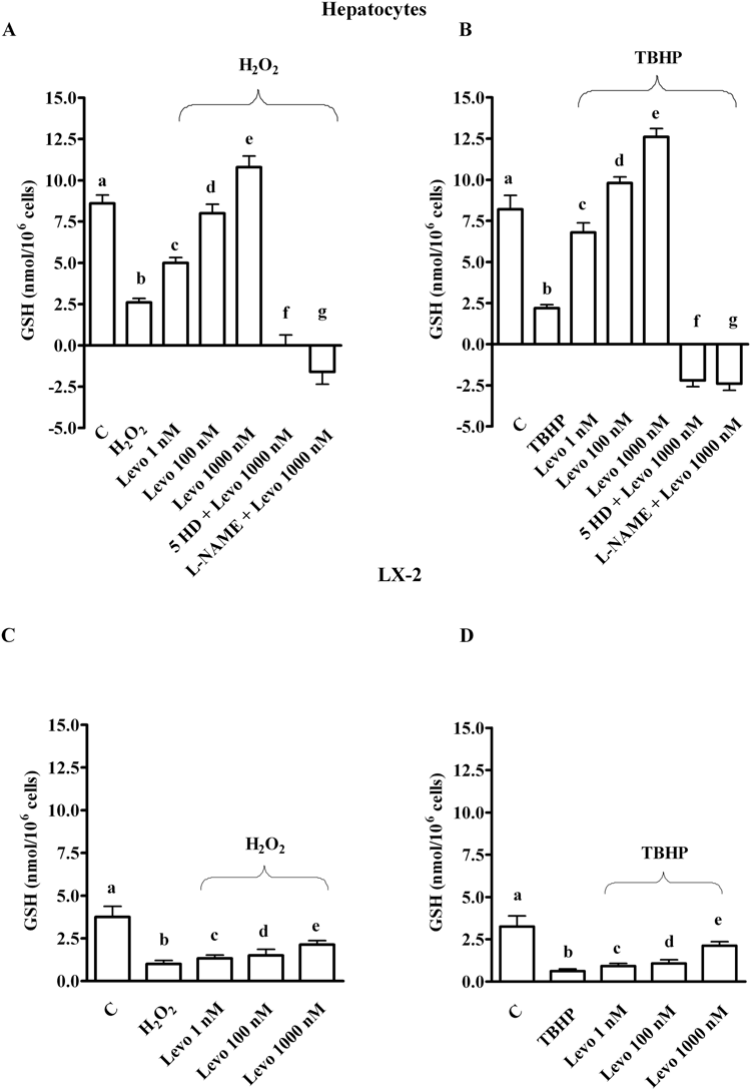
Effects of levosimendan on glutathione content in hepatocytes and LX-2. In A and B, b, c, e, f, g *P*<0.05 vs a; c, d, e, f, g *P*<0.05 vs b; d, e *P*<0.05 vs c; d, f, g *P*<0.05 vs e. In C and D, b, c, d *P*<0.05 vs a; e *P*<0.05 vs b. In D, e *P*<0.05 vs c, d. GSH = glutathione. Other abbreviations are as in Figs 1 and 2. The results obtained in hepatocytes and LX-2 are expressed as means of 5 or 4 independent experiments (%) ± SD (indicated by the bars), respectively.

As shown in Fig 1, oxidative stress generated by both 200 μM H_2_O_2_ and 250 μM tert-butyl hydroperoxide (TBHP) reduced survival in both hepatocytes and LX-2. In addition, an increase of ROS generation and a decrease of GSH were observed (Figs 2 and 3). Pre-treatment of hepatocytes with levosimendan dose-dependently counteracted the effects of oxidative stress and improved survival rate (Figs 1–3). It is notable that in LX-2, in spite of ROS reduction the percentage of survival and GSH content were lower than control values even for levosimendan given at the highest dose (Figs 1–3). Since the effects of TBHP were not different from those caused by H_2_O_2_ all following experiments were performed by using hydrogen peroxide only.

Since eNOS/iNOS and related NO release have been reported to play a role in protection/damage against oxidative stress [20–22], we next examined the effects of levosimendan on NO production and the involvement of those NOS isoforms in the protection against peroxidative injuries in liver cells.

**Fig 4:**
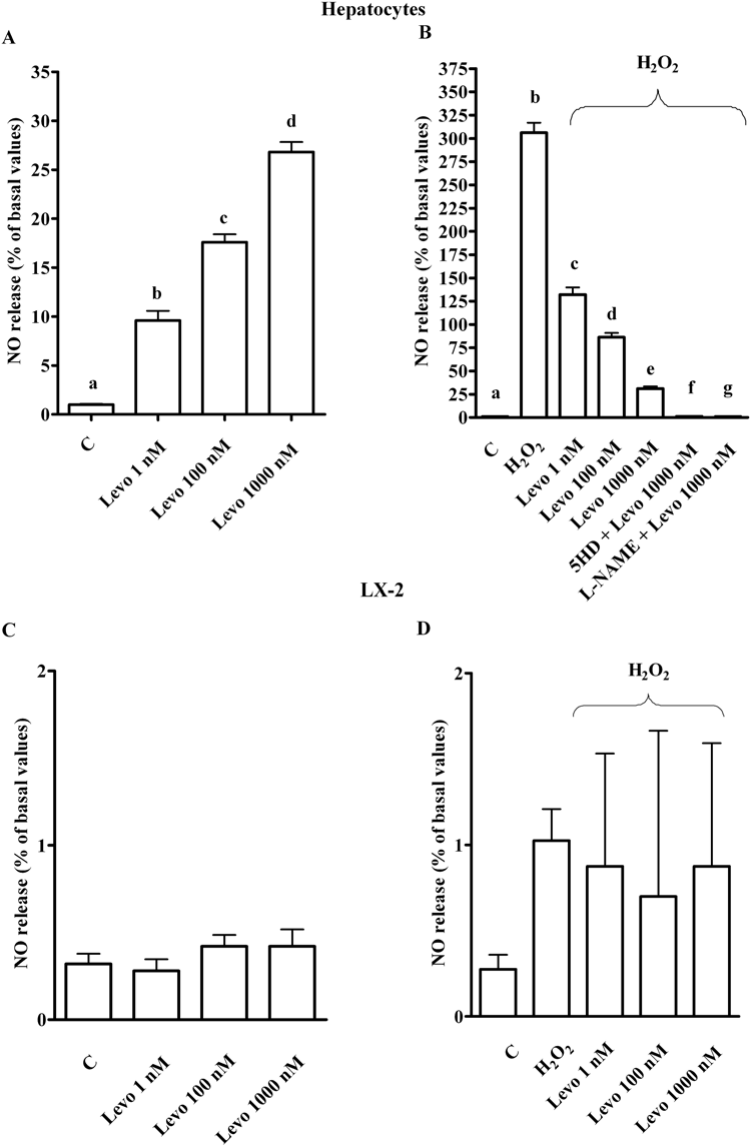
Effects of levosimendan on nitric oxide release in hepatocytes and LX-2. In A, hepatocytes in physiological condition. b, c, d *P* <0.05 vs a; c, d *P* <0.05 vs b; d *P* <0.05 vs c. In B and C, hepatocytes in peroxidative conditions. b, c, d, e *P* <0.05 vs a; c, d, e, f, g *P* <0.05 vs b; d, e *P* <0.05 vs c; d, f, g *P* <0.05 vs e. In C and D, in LX-2. NO = nitric oxide. Other abbreviations are as in Figs 1–3. The results obtained in hepatocytes and LX-2 are expressed as means of 5 and 4 independent experiments (%) ± SD (indicated by the bars), respectively.

**Fig 5:**
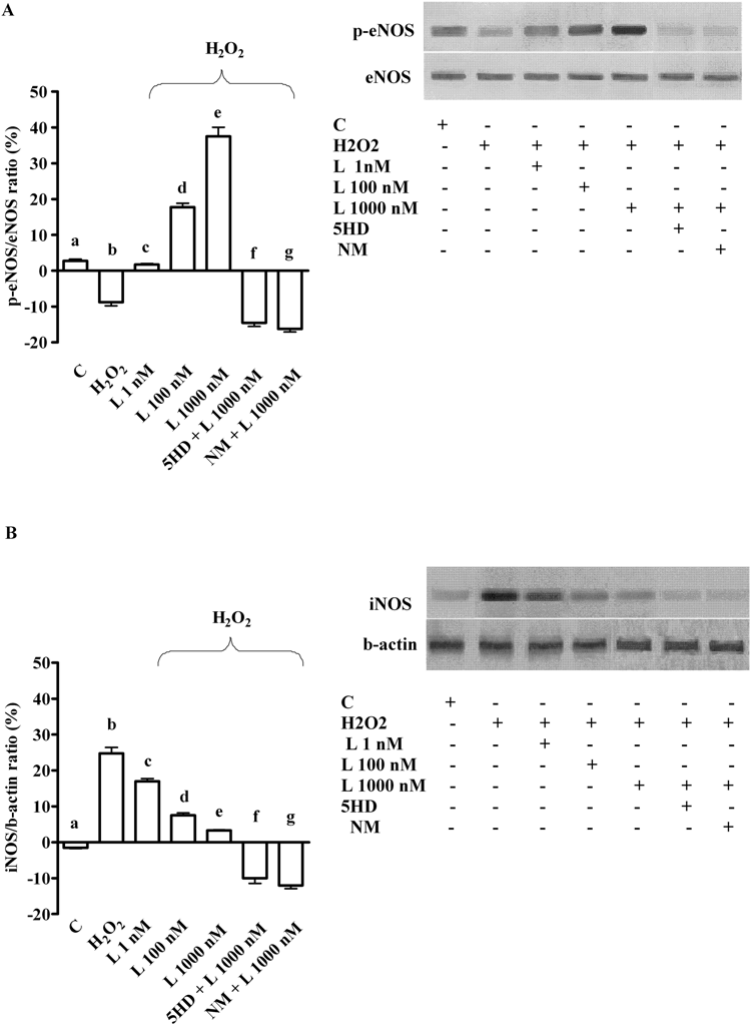
Effects of levosimendan on eNOS (A) and iNOS activation (B) in hepatocytes subjected to peroxidation. In A and B, densitometric analysis of p-eNOS and iNOS and an example of lanes taken in one of 5 different experiments performed for each experimental protocol. eNOS = endothelial nitric oxide isoform; iNOS = inducible nitric oxide isoform; L = levosimendan; NM = L-NAME. The other abbreviations are as in Figs 1–4. In A, b, d, e, f, g *P* <0.05 vs a; c, d, e, f, g *P* <0.05 vs b; d, e p<0.05 vs c; d, f, g *P* <0.05 vs e. In B, b, c, d, e, f, g *P* <0.05 vs a; c, d, e, f, g *P* <0.05 vs b; d, e *P* <0.05 vs c; d, f, g *P* <0.05 vs e. The results of densitometric analysis are expressed as means of 5 independent experiments (%) ± SD (indicated by the bars).

As shown in Fig 4A, in physiologic condition levosimendan dose-dependently increased NO release in hepatocytes, which confirmed previous findings about the involvement of NO in the effects of levosimendan [23]. Moreover, pre-treatment of hepatocytes with 200 μM H_2_O_2_ strongly increased NO release (Fig 4B), an effect that was accompanied by p-eNOS inhibition and iNOS activation (Fig 5). As shown in Fig 4B, in peroxidative conditions levosimendan was able to counteract NO release by restoring eNOS/iNOS ratio in hepatocytes (Fig 5).

Different results were obtained in LX-2 where NO release was not affected by levosimendan, neither in physiologic or peroxidative conditions (Fig 4C and 4D).

### Effects of levosimendan on apoptosis and autophagy in hepatocytes and LX-2

**Fig 6:**
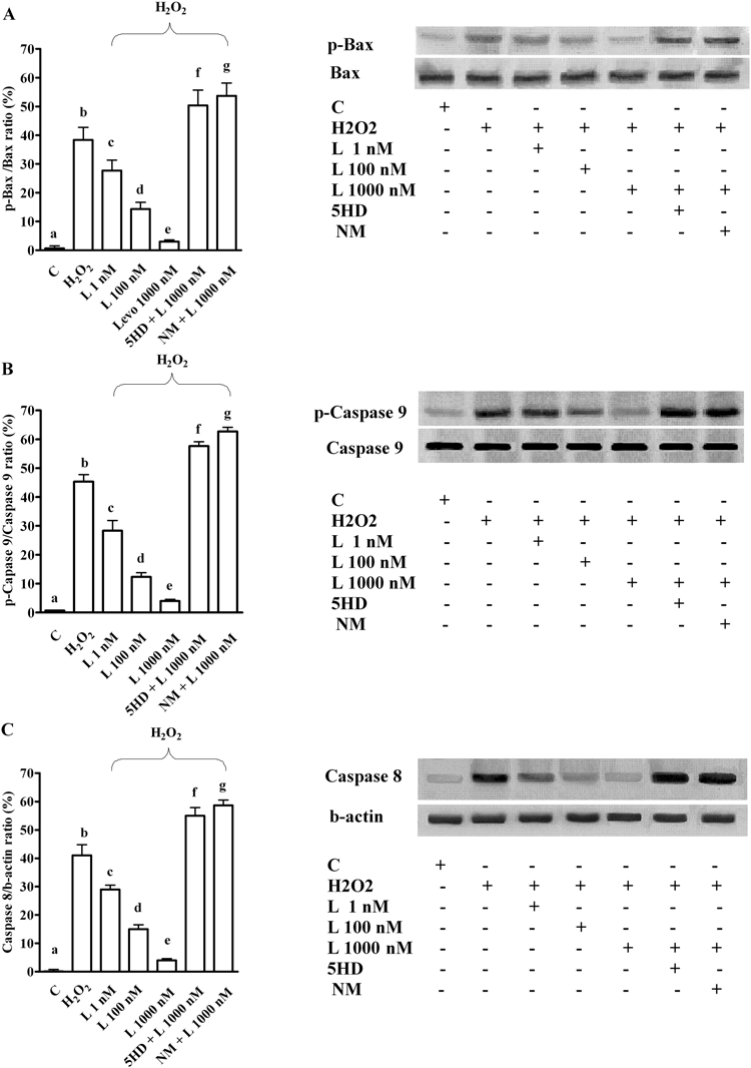
Effects of levosimendan on apoptosis caused by peroxidation in hepatocytes. In A, B and C, effects of levosimendan on p-Bax, p-Caspase 9 and Caspase 8, respectively. Densitometric analysis and an example of lanes taken in one of 5 different experiments performed for each experimental protocol are reported. Abbreviations are as in Figs 1–5. b, c, d, e, f, g *P* <0.05 vs a; c, d, e, f, g *P* <0.05 vs b; d, e *P* <0.05 vs c; d, f, g *P* <0.05 vs e. The results of densitometric analysis are expressed as means of 5 independent experiments (%) ± SD (indicated by the bars).

**Fig 7:**
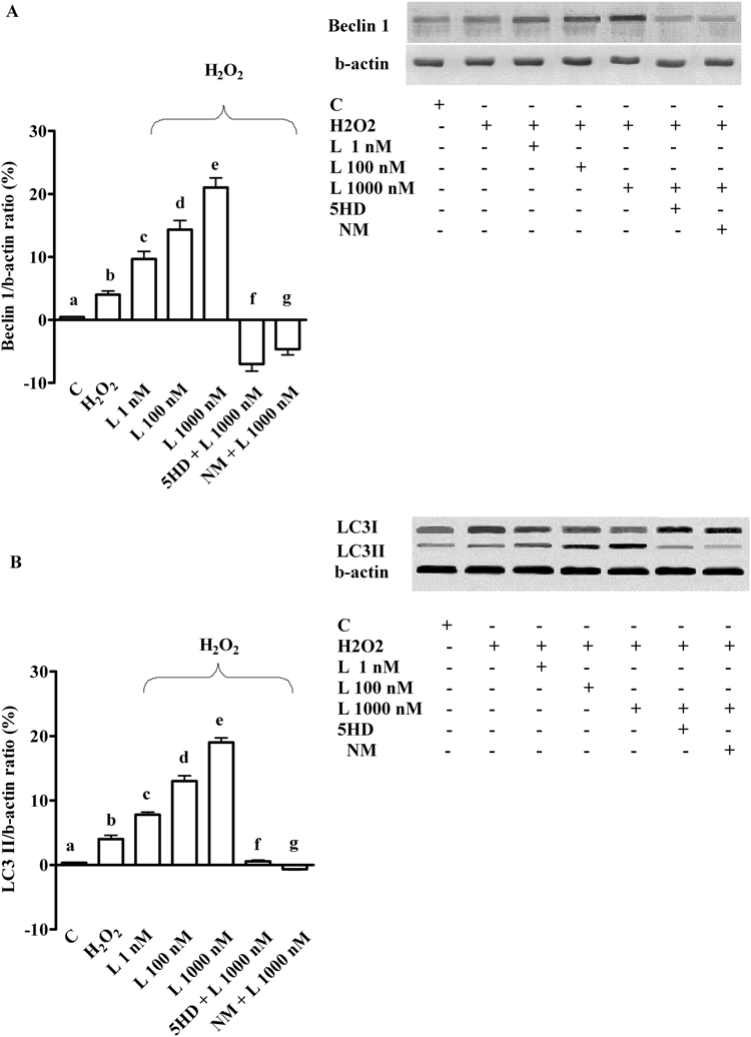
Effects of levosimendan on autophagy caused by peroxidation in hepatocytes. In A and B, effects of levosimendan on Beclin 1 and LC3I/II, respectively. Densitometric analysis of Beclin 1 and of LC3II and an example of lanes taken in one of the 5 different experiments performed for each experimental protocol are reported. Abbreviations are as in Figs 1–6. In A, b, c, d, e, f, g *P* <0.05 vs a; c, d, e, f, g *P* <0.05 vs b; d, e *P* <0.05 vs c; d, f, g *P* <0.05 vs e. In B, b, c, d, e *P* <0.05 vs a; c, d, e, f, g *P* <0.05 vs b; d, e *P* <0.05 vs c; d, f, g *P* <0.05 vs e. The results of densitometric analysis are expressed as means of 5 independent experiments (%) ± SD (indicated by the bars).

**Fig 8:**
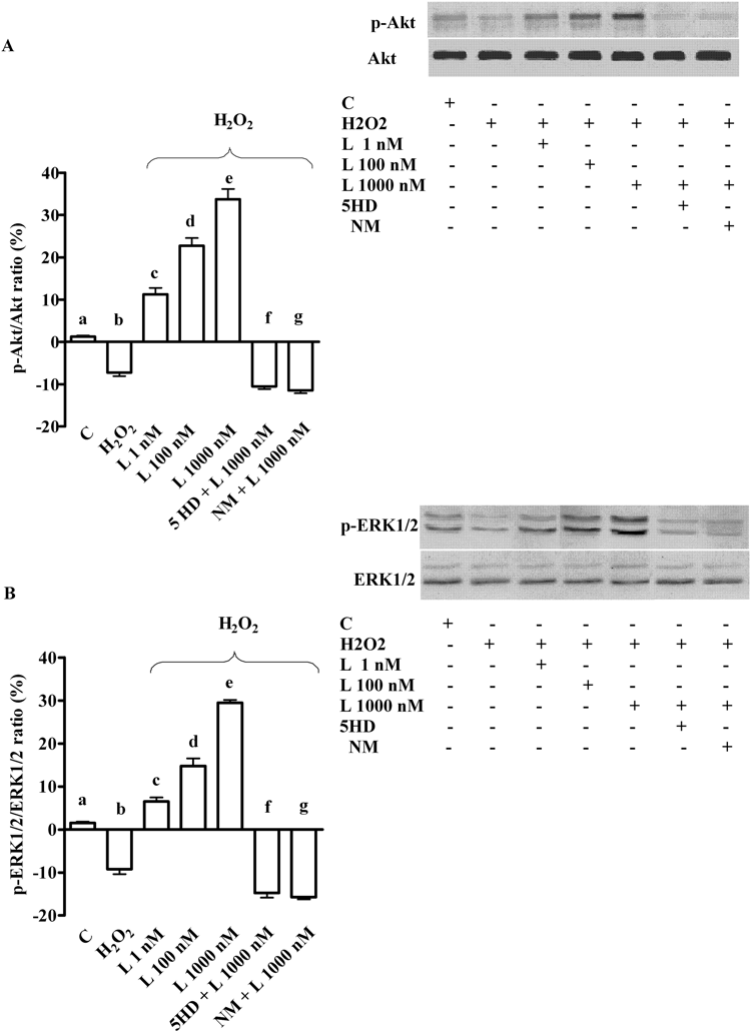
Effects of levosimendan on cell survival signalling in hepatocytes subjected to peroxidation. In A and B, effects of levosimendan on p-Akt and p-ERK1/2, respectively. Densitometric analysis and an example of lanes taken in one of the 5 different experiments performed for each experimental protocol are reported, as well. Abbreviations are as in previous Figures. b, c, d, e, f, g *P* <0.05 vs a; c, d, e, f, g *P* <0.05 vs b; d, e *P* <0.05 vs c; d, f, g *P* <0.05 vs e. The results of densitometric analysis are expressed as means of 5 independent experiments (%) ± SD (indicated by the bars).

The pre-treatment of hepatocytes with levosimendan prevented apoptosis activation caused by H_2_O_2_ while increasing autophagy and cell survival signalling (Figs 6–8).

After the pan-caspases inhibitor (Z-VAD.FMK, 25 mM), p-Bax was reduced (Table 1) whereas cell survival (Fig 1A), the autophagic pathway and Akt phosphorylation were increased (Table 1). Moreover, after Z-VAD.FMK, the effects of levosimendan on cell viability, autophagy, and survival signalling were increased (Fig 1A; Table 1).

**Table 1:** Densitometric analysis of apoptosis, autophagy and survival signalling activated by various agents in hepatocytes treated with H_2_O_2_.

Data	p-Bax/Bax	Beclin1/b actin	LC3II/b actin	p-Akt/Akt
**C**	0.4 ± 0.8	0.4 ± 0.2	0.34 ± 0.2	1.2 ± 0.4
**H2O2**	47.8 ± 7*	3.6 ± 1.1*	4 ± 1.5*	-6.6 ± 2*
**L + H2O2**	2.8 ± 0.8* †	21.2 ± 2.6 * †	17.2 ± 2* †	33 ± 5* †
**Z-VAD + H2O2**	-7.8 ± 1.3* †	6.8 ± 1.3* †	10.4 ± 1.1* †	15 ± 2.7* †
**Z-VAD + L+ H2O2**	-8.2 ± 1.5* ††	28.8 ± 3.7* ††	25 ± 3.1* ††	42 ± 5.1* ††
**5HD+Z-VAD + L+ H2O2**	0.4 ± 0.9††	-5.2 ± 1.3* ††	0.6 ± 0.5* ††	-5 ± 1.6* ††
**NM+Z-VAD + L + H2O2**	1 ± 0.7††	-6.2 ± 1.3* ††	-0.7 ± 0.5* ††	-8.8 ± 0.8* ††
**C**	0.3 ± 0.7	0.5 ± 0.1	0.38± 0.2	1.6 ± 0.5
**H2O2**	48.2 ± 6.6*	3 ± 1*	4.2 ± 0.8*	-7.2 ± 1.4*
**L+ H2O2**	2.4 ± 0.5* †	22.8 ± 2.6* †	17.6 ± 2.4* †	34.4 ± 5.4* †
**RAPA + H2O2**	3.8 ± 1.3* †	33.6 ± 2.5* †	35.2 ± 3.2* †	42 ± 3.9* †
**RAPA +L + H2O2**	-8.8 ± 1.9* ††	46.4 ± 2.8* ††	53.8 ± 3* ††	57 ± 4.1* ††
**5HD+RAPA+ L+ H2O2**	11.2 ± 1.9* ††	1.8 ± 0.8* ††	1.4 ± 0.5* ††	-1.8 ± 0.8* ††
**NM+RAPA+ L+ H2O2**	9.6 ± 1.5* ††	-1.4 ± 1.6* ††	-2.6 ± 1.1* ††	-6.2 ± 0.8* ††
**C**	0.3 ± 0.7	0.6 ± 0.08	0.36 ± 0.09	1.8 ± 0.8
**H2O2**	49.4 ± 6.8*	2.8 ± 0.8*	3.8 ± 0.8*	-7.8 ± 0.8*
**L+ H2O2**	2 ± 0.7* †	24 ± 2.9* †	18 ± 2.2* †	35.6 ± 5.1* †
**3MA + H2O2**	52.8 ± 7.8* †	-7.4 ± 1.1* †	-9.8 ± 1.8* †	-10.8 ± 1.3* †
**3MA + L + H2O2**	52.2 ± 5.2* ††	-7.6 ± 1.5* ††	-10.4 ± 1.6* ††	-11.4 ± 11.5* ††
**5HD+3MA L+ H2O2**	62.8 ± 7* ††	-14.2 ± 1.9* ††	-14.4 ± 1.1* ††	-15.2 ± 0.8* ††
**NM+3MA + L+ H2O2**	64.4 ± 7.5* ††	-14.4 ± 3* ††	-15.2 ± 1.8* ††	-15.4 ± 1.1* ††

Data are means (%) ± SD of 5 independent experiments. C: control; L = levosimendan (1 μM); Z-VAD = Benzyloxycarbonyl-Val-Ala-Asp (OMe) fluoromethylketone (25 mM); 5HD: 5 hydroxydecanoate (1 μM); NM = L-NAME (10 mM); RAPA: rapamicyn (100 nM); 3MA: 3 metyladenine (10 mM).

* *P*<0.001 *vs*. control;

^†^ *P*<0.001 *vs*. H_2_O_2_;

^††^ *P*<0.001 *vs*. L + H_2_O_2_

After autophagy inhibition through 3 metyladenine (3-MA, 10 mM), the apoptotic pathway was activated, whereas survival signalling and cell viability were reduced (Table 1; Fig 1A). Moreover, the effects of levosimendan were impaired and the antiapoptotic action elicited by levosimendan was prevented.

As reported in Table 1 and Fig 1A, the autophagy activator, rapamycin (100 nM) in hepatocytes exerted similar effects to those of levosimendan on apoptosis, cell viability and survival signalling. In addition, the co-stimulation of rapamycin and levosimendan amplified the effects of both agents given alone.

Those findings highlighted the existence of a cross talk between apoptosis and autophagy, which would play a role in cell survival and in eliciting the protective effects of levosimendan against peroxidation in hepatocytes.

**Fig 9:**
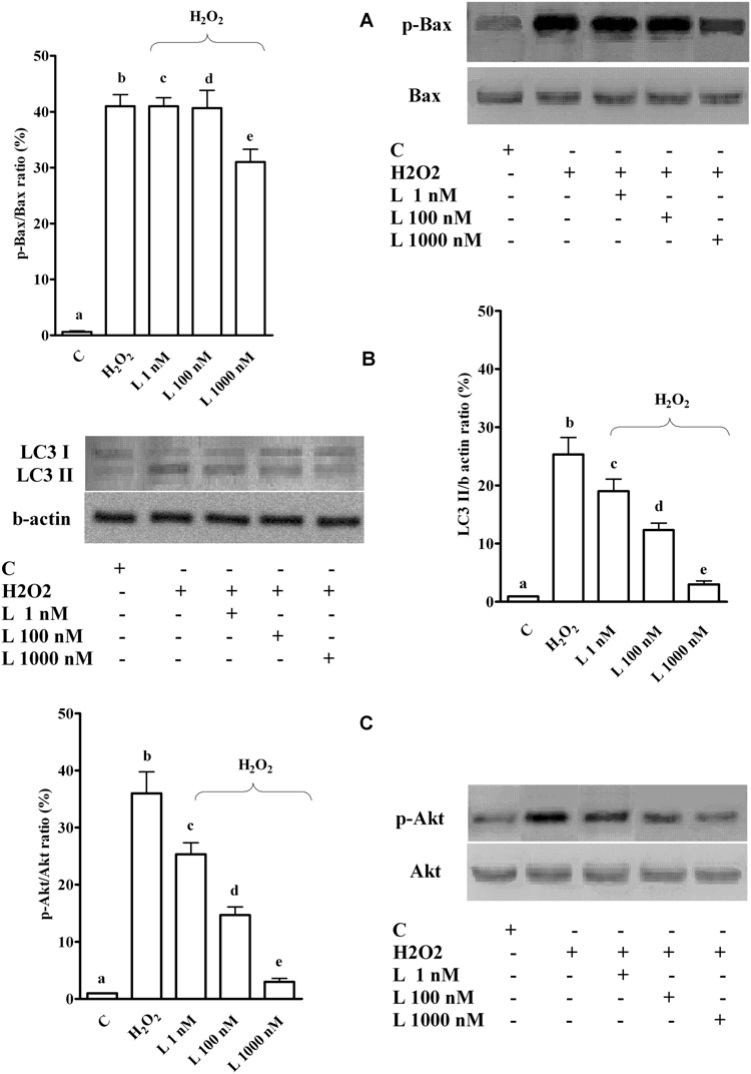
Effects of levosimendan on apoptosis, autophagy and cell survival signalling in LX-2 subjected to peroxidation. In A, B and C, effects of levosimendan on p-Bax, LC3I/II and p-Akt, respectively. Densitometric analysis of p-Bax, LC3II and p-Akt and an example of lanes taken in one of the 3 different experiments performed for each experimental protocol are reported. Abbreviations are as in Figs 1–7. In A, b, c, d, e *P* <0.05 vs a; e *P* <0.05 vs b, c, d. In B, b, c, d, e *P* <0.05 vs a; c, d, e *P* <0.05 vs b; d, e *P* <0.05 vs c; e *P* <0.05 vs d. In C, b, c, d, e *P* <0.05 vs a; c, d, e *P* <0.05 vs b; d, e *P* <0.05 vs c; e *P* <0.05 vs d. The results of densitometric analysis are expressed as means of 3 independent experiments (%) ± SD (indicated by the bars).

In LX-2, levosimendan dose-dependently reduced autophagy and Akt activation caused by H_2_O_2_ (Fig 9B and 9C). Regarding apoptosis, a slight reduction of p-Bax was observed only at the highest dose levosimendan (Fig 9A). However, p-Bax was still higher than control values.

### Effects of levosimendan on mitochondrial function in hepatocytes and LX-2 subjected to peroxidation

**Fig 10:**
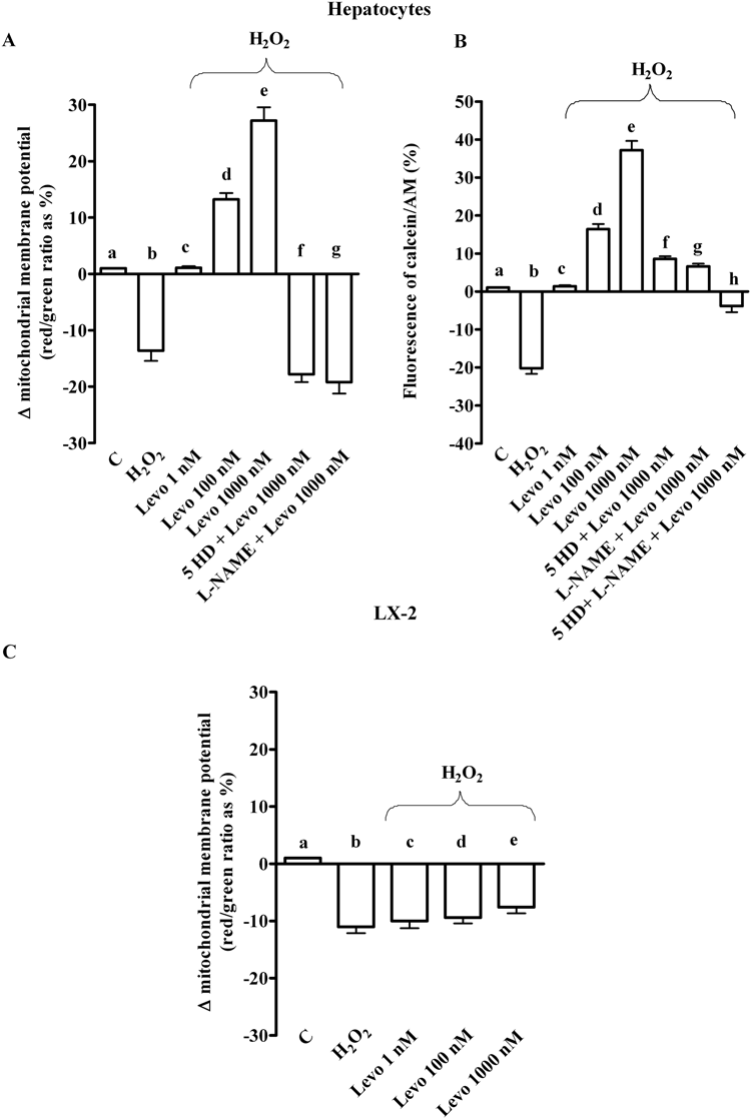
Effects of levosimendan on mitochondrial function in hepatocytes and LX-2 subjected to peroxidation. In A and B, effects of levosimendan on mitochondrial membrane potential and permeability transition pore opening in hepatocytes. b, d, e, f, g *P* <0.05 vs a; c, d, e, f, g *P* <0.05 vs b; e *P* <0.05 vs c; d, f, g *P* <0.05 vs e. In B, h *P* <0.05 vs a, b, e, f, g. In C effects of levosimendan on mitochondrial membrane potential in LX-2. b, c, d, e *P* <0.05 vs a. In C, e *P* <0.05 vs c. Abbreviations are as in previous Figures. The results obtained in hepatocytes and LX-2 are expressed as means of 5 and 3 independent experiments (%) ± SD (indicated by the bars), respectively.

As shown in Fig 10A and 10B, 200 μM H_2_O_2_ caused the collapse of mitochondrial membrane potential and the reduction of mitochondria-trapped calcein intensity in hepatocytes, an effect which was dose-dependently prevented by levosimendan.

Different results were obtained in LX-2, where levosimendan did not prevent any changes in mitochondrial membrane potential at any doses (Fig 10C).

### Role of mitoK_ATP_ channels and NO in the protective effects elicited by levosimendan in hepatocytes

In hepatocytes, the effects of levosimendan on NO release, cell survival, apoptosis and autophagy were abolished by the mitochondrial K_ATP_ channels blocker, 5 hydroxydecanoate (5HD; 1 μM) and the NOS inhibitor, Nω-nitro-L-arginine methyl ester (L-NAME; 10 mM; Figs 1A and 4B, Figs 5–8). These findings are in agreement with those observed in the *in vivo* study about the protection exerted by levosimendan in liver, as well as in heart and kidney [5–8].

In addition, while the pre-treatment of cells with L-NAME potentiated the effects of levosimendan on ROS generation, in cells pre-treated with 5HD, the ROS generation was augmented (Fig 2 and 2B). It is notable that both blockers inhibited the response of levosimendan on GSH levels (Fig 3A and 3B) and on preserving mitochondrial membrane potential (Fig 10A). Moreover, both 5HD and L-NAME reduced, when given separately, or prevented when given in co-administration, the effect of levosimendan on mitochondrial transition pore (MPTP) opening (Fig 10B). Taken together those results highlighted a rather complex role played by NO in the modulation of cell damage, whereas mitochondrial K_ATP_ channels opening would be beneficial for cell survival.

It is also notable that both 5HD and L-NAME reverted the beneficial effects exerted by the pan-caspases inhibitor (Z-VAD.FMK, 25 mM) and levosimendan in co-stimulation (Table 1). Furthermore, in cells pre-treated with the autophagy activator, rapamycin (100 nM), the effects of levosimendan in presence of 5HD and L-NAME on autophagy and p-Akt were abolished, whereas those on apoptosis were increased. Finally, in presence of 5HD and L-NAME the effects of the autophagy inhibitor, 3-methyladenine (3-MA, 10 mM) on apoptosis were increased whereas p-Akt was further inhibited (Table 1).

## Discussion

In this study, we found that levosimendan protects primary rat hepatocytes against hydrogen peroxide-induced cell death by inhibition of ROS production and preservation of antioxidant systems. Moreover, our results showed that in those cells levosimendan can modulate apoptosis/autophagy interplay and activate cell survival pathways against oxidative injuries. Those protective effects are related to mitoK_ATP_ channels opening and NO production modulation. Also the findings obtained in LX-2 would suggest a role for levosimendan as a protective agent against human stellate hepatic cells (HSC) activation and against the possible fibrotic evolution.

Oxidative stress contributes to a wide spectrum of diseases and modulates acute and chronic cellular injury in the liver [24–26]. The damage created by oxidative stress affects hepatocytes by not only inducing inflammation and necrosis but also apoptosis [27]. In addition, recent studies suggest that autophagy is another distinct form of cell death that hepatocytes sustain when exposed to oxidative stress; however, cells undergoing autophagy are not committed irreversibly to death and autophagy could improve cell survival by acting as a physiologically protective system [9, 28].

In the present study, 30 min pre-treatment with levosimendan at similar doses as the ones previously used [5–8, 23] dose-dependently prevented the loss of cell viability and reduced ROS release caused by either hydrogen peroxide or superoxide anion donor, TBHP, while preventing the GSH fall. It is notable that ROS release was detected thorough DCF-DA, which is widely used for intracellular ROS quantification in both hepatic cells and LX-2 [29–31]. Those effects were accompanied by apoptosis inhibition and by an increase of the proautophagic Beclin 1 and LC3II and of the levels of phosphorylation of ERK1/2 and PI3K/Akt, members of the so-called reperfusion injury salvage kinase (RISK) pathway, which are critically involved in hepatocytes survival, too [32].

By using apoptosis and autophagy inhibitors, the present study provided evidence in hepatocytes that these processes can interact with each other in modulating cell survival and protection against oxidative injuries. Moreover, as previously shown in cardiomyocytes, levosimendan was found to play a role as an inhibitor of hydrogen peroxide-induced cell death through the modulation of apoptosis/autophagy cross-talk.

Hence, in hepatocytes pre-treated with the pan-caspase inhibitor, Z-VAD.FMK [6, 11], the activation of autophagic markers and of the members of Akt was increased. In addition, those effects were augmented by further administration of levosimendan. On the contrary, the use of the blocker of the early stages of autophagy, 3-MA [11], was able to sensitize cells to H_2_O_2_-induced cell death through increased apoptosis. It is notable that in hepatocytes pre-treated with 3-MA, both LC3II and Beclin 1 were inhibited, which would be consistent with strong autophagy process blocking. Similar effects of 3-MA were obtained in cardiomyocytes subjected to oxidative stress too [6]. Besides, 3-MA has been widely used for autophagy inhibition in hepatocytes, as well [33].

Moreover, after 3-MA, the effects of levosimendan on cell viability, survival pathway and apoptosis were prevented. These findings are in agreement with previous reports showing that disabled autophagy reduces cell viability through a final pathway involving biochemical features of apoptosis in various cellular models, among which hepatocytes, as well [6].

That levosimendan could exert its protective effects in hepatocytes subjected to oxidative injuries through modulation of apoptosis/autophagy interplay was further confirmed by experiments performed in presence of the pro-autophagic factor, rapamycin [6], which increased cell viability and survival signalling while inhibiting apoptosis, and increased the response of cells to levosimendan.

It is widely accepted that in hepatocytes the loss of the proton gradient and electrical potential across the mitochondrial membrane can also lead to cell death through apoptosis activation [34]. In addition, the mitoK_ATP_ have been reported to have critical effects in regulating mitochondrial volume, as well as function in hepatocytes, too [35, 36]. In the present study, levosimendan counteracted the loss of mitochondrial membrane potential and MPTP opening caused by hydrogen peroxide, through mechanisms involving mitoK_ATP_ channels activation, as shown through experiments performed in presence of the mitoK_ATP_ inhibitor, 5HD.

Moreover, levosimendan was found for the first time to modulate eNOS/iNOS-dependent NO release in hepatocytes. Hence, while under “physiological” conditions levosimendan caused a dose- and eNOS-dependent NO release, during peroxidative stress it reduced the NO release. Furthermore, this effect was accompanied by the inhibition of the iNOS activation and the prevention of the eNOS inhibition.

The effects of NO in liver protection against peroxidative injuries have been the subject of studies in the last years. Hence, NO has been found to exert beneficial effects through increased hepatic ATP levels, reduced oxidative damage, and prevention of the reduction of antioxidants such as glutathione [37].

Studies have demonstrated that NO can affect cellular decisions of life and death by either turning on or shutting off apoptotic pathways, suggesting that NO can function differently depending on the dose and duration of exposure [37]. In this respect it is notable that while NO produced in low concentration, as in the case of eNOS activation, would act as a messenger and cytoprotective factor *via* direct interactions with transition metals and other free radicals [38, 39], it could increase reactive nitrose species formation and cause cellular death when over-secreted by iNOS [6, 40, 41]. Thus, imbalance in normal cellular conditions of the various isoforms of NOS and in NO release would result in profound disturbances leading to increased hepatocytes injuries [42].

Although not clearly demonstrated, it could be speculated that findings about NO release in oxidative conditions could be related to the blocking of the iNOS subtype and the keeping of p-eNOS by levosimendan.

Furthermore, the protection exerted by levosimendan through eNOS modulation could be related to the prevention of eNOS “uncoupling”. In this respect it is worth noting that eNOS has recently been reported to be a redox “hub”, contributing to the regulation of intracellular redox homeostasis through interaction with GSH-related pathways. Hence, changes of GSH have been reported to cause eNOS “uncoupling”, which would trigger ROS production from the oxygenase domain [43].

In the present study, levosimendan was found to prevent GSH reduction and ROS generation and the eNOS inhibition caused by peroxidative stress in hepatocytes. Thus, the modulation of eNOS function caused by levosimendan could be speculated to be related to glutathione-dependent prevention of eNOS “uncoupling”. By this way levosimendan would preserve physiological and beneficial NO release in hepatocytes following oxidative stress by both preventing further ROS production by “uncoupled” eNOS and by iNOS inhibition. It is notable that in presence of the non-selective NOS blocker, L-NAME, the detrimental effects of peroxidation on hepatocytes were increased and the response of cells to levosimendan abolished or reduced, which would be in agreement with above observations. However, further experiments performed by examining the different role played by various NOS isoforms could be useful to better address this issue.

The present results add new information about the role of autophagy in liver protection against oxidative stress. Indeed, in livers subjected to ischemia/reperfusion injuries, autophagy would mainly act as a prosurvival mechanism allowing the cell to cope with hypoxia [44]. Our results are in line with those observations. Hence, as reported above in presence of 3-MA, cell viability was reduced and apoptosis activated, which was suggestive of the existence of a role of autophagy as cell survival promoter through inhibition of apoptosis pathway in liver.

Also, the results obtained in LX-2 would support the beneficial use of levosimendan against peroxidative injuries. Indeed, the activation of HSC by ROS has been widely accepted to be a common event in the initiation and progression of liver fibrosis. Thus, ROS can induce HSC proliferation and collagen synthesis, which is aggravated by depletion of antioxidants that can be observed in liver diseases [22, 31]. The activation of ERK1/2 and PI3K/Akt would also be involved in the intracellular signalling leading to HSC activation and transformation into a myofibroblast-like phenotype [22, 45]. Furthermore, autophagy that could be activated by oxidative stress in LX-2, has also been reported to play a profibrogenic role [46].

The effects of levosimendan in LX-2 were partially different from those found in hepatocytes. Firstly, levosimendan was only able to reduce ROS generation and the collapse of GSH levels but only at the highest dose. In addition, differently from what was observed in hepatocytes, in LX-2 levosimendan did not prevent either the fall of mitochondrial membrane potential or apoptosis caused by hydrogen peroxide, which could explain the absence of significant effects on cell survival. Moreover, those effects were accompanied by dose-dependent inhibition of autophagy and of Akt activation. Thus, those results would suggest a role for levosimendan as inhibitor of LX-2 activation through reduction of peroxidative stimuli and autophagic process.

Furthermore, no significant effects were observed on NO release in LX-2. Reported data are quite contradictory, showing either no significant NO production or increased NO release in HSC [21, 22], depending on the experimental condition. While eNOS-derived NO by hepatocytes could exert paracrine effects on adjacent HSC by inhibiting the proliferation and migration, opposite effects would be played by iNOS-related NO release [47]. Thus, the protective effects elicited by levosimendan on LX-2 could also be due to eNOS/iNOS modulation in hepatocytes.

Future experiments would be useful to clarify this issue, as well as the role of autophagy as a modulator of apoptotic signal in LX-2 and in the progression of fibrotic process.

All the above considerations, together with data about the effects of levosimendan [48–50], are of particular interest to clinical practice. Hence, the liver is susceptible to numerous conditions associated with peroxidative stress, such as extensive liver resections and transplantation. In all those conditions mitochondria play key roles in energy production and in modulation of programmed cell death [37]. In particular, it is assumed that mitoK_ATP_ may be able to prevent long-term opening of MPTP, thus preserving the integrity of the mitochondria and ensuring a better cellular energy status and pro-survival actions of hepatocytes. In this context levosimendan, by acting through modulation of mitochondria functioning and cell survival, could be hypothesized as a therapeutic agent for the prevention/reduction of liver peroxidative injuries. Additionally, levosimendan could also counteract fibrotic transformation induced by oxidative stress through mechanisms involving apoptosis/autophagy interplay in HSC. Finally, the effects of levosimendan on hepatic function has been recently explored in critically ill patients with promising results [51–54] There is, therefore, a strong rationale to run proper clinical trials to assess the role and clinical relevance of levosimendan as therapeutic agent for the treatment or prevention of liver ischemia/reperfusion injuries.
